# Pneumococcal Serotypes Recovered from Health Children and Their Possible Association with Risk Factor in Istanbul, Turkey

**Published:** 2009-06

**Authors:** Ferhat Cekmez, Ferhan Karademir, I. Asya Tanju, Cihan Meral, Ozgur Pirgon, Mustafa Ozyurt, Ismail Gocmen

**Affiliations:** 1 *Department of Pediatrics, GATA Medical Faculty, Istanbul, Turkey;*; 2 *Department of Microbiology, GATA, Medical Faculty, Istanbul, Turkey*

**Keywords:** *Streptococcus pneumoniae*, colonization, vaccine

## Abstract

*Streptococcus pneumonia* is an increasing problem worldwide and nasopharyngeal colonization plays an important role in pneumococcal infections. The aims of this study were to assess the nasopharyngeal colonization rate, investigate the risk factors for nasopharyngeal colonization with *S. pneumonia*, serogroup and penicillin susceptibility patterns of *Streptococcus pneumoniae* strains isolated from healthy children. A single swab was obtained over the nasopharyngeal walls of the 500 healthy children, of which 25 (5%) were found to be carriers. The carrier rate was significantly higher in the seven-nine years-old age group. Upper respiratory tract infection within the last month (OR=1.1, p>0.001), day-care attendance (OR=3.1, p: 0.02), and the presence of more than five people living in the house of the child (OR=0.2, p: 0.003) have been determined to be risk factors for *S. pneumoniae* carriage. The most prevalent serogroups in descending order were 9, 19, 23, 6, 10 and 18 and these are in accordance with conjugated pneumococcal vaccine (PCV7). No penicillin-resistant *S. pneumoniae* were obtained. Conclusion: after conjugated vaccine we have seen benefits of vaccine in especially colonization rate and penicillin-resistance.

## INTRODUCTION

*Streptococcus pneumoniae* is one of the most important agents of serious infections observed in children. In recent years, emergence of penicillin-resistant *S. pneumoniae* strains has made it difficult to treat the infections caused by this organism (2). Although nasopharyngeal colonization plays an important role for infections of *S. pneumoniae*, the factors affecting the nasopharyngeal colonization with or without penicillin-resistant *S. pneumoniae* have not been understood sufficiently. Various risk factors for nasopharyngeal colonization with penicillin susceptible or resistant *S. pneumoniae* have been reported (3, 17). Some of these factors such as living conditions and genetic traits may be related to regional differences (5, 19). These differences may explain why the incidence of invasive pneumococcal disease in children in the developing world is several times higher than in industrialized countries, and regional differences for penicillin resistance (8).

In Turkey, a developing country, pneumococcal infections and penicillin resistance to *S. pneumoniae* are an important public health problem (15, 22). In developing countries, investigation of risk factors for penicillin-resistant *S. pneumonia* may help in understanding the pathogenesis of pneumococcal infections and preventing the emergence of resistant strains. The aims of this study were to assess the nasopharyngeal colonisation rate, investigate the risk factors for nasopharyngeal colonization with *S. pneumonia,* serogroup and penicillin susceptibility patterns of *Streptococcus pneumoniae* strains isolated from healthy children.

## MATERIALS AND METHODS

### Study groups

500 Children aged between 1 months and 12 years without evidence of infection in GATA Haydarpasa Military Hospital, Department of Paediatrics, between January, 2007 and January 2008, were enrolled in the study. This study group was divided into four groups according to ages 0-3, 4-6, 7-9, 10-12 years- old age respectively.

**Investigation of risk factors for nasopharyngeal carriage of *S. pneumoniae*.** Initially, a questionnaire was given to the parents or guardians about potential risk factors that may affect *S. pneumoniae* carriage and resistant strain carriage. Through this questionnaire, information was collected about the following parameters: (a) age; (b) gender; (c) the child’s upper respiratory tract infection history in the last month; (d) the use of at least one cure of antibiotic in the last three months; (e) the use of at least one cure of antibiotic by family members in the last three months; (f) day-care attendance; (g) the presence of more than five people living in the house of the child; (h) the presence of younger than five-years-old siblings; (i) breast-feeding history (j) parental smoking history; (k) family’s monthly income. The data obtained from the questionnaire were confirmed by available medical records. Informed consent from parents or guardians was obtained prior to the study.

### Bacterial culture

A single swab was obtained over the posterior nasopharyngeal walls of the 500 apparently healthy children. Each swab was immediately inoculated on trypticase soy agar plates (Oxoid Ltd., Basingstoke, UK) supplemented with 5% defibrinated sheep’s blood. After incubation overnight at 37 °C in 5% CO_2_ atmosphere, characteristic α-haemolytic colonies were isolated and identified as *S. pneumoniae* using optochin susceptibility (5 U disk) and bile solubility tests.

### Susceptibility test

The disk diffusion method was performed on Mueller–Hinton agar (Oxoid Ltd., Basingstoke, UK) with 5% sheep’s blood in accordance with the guidelines of the National Committee for Clinical Laboratory Standards (11). Penicillin resistance was screened with 1 μg oxacillin disk (Oxoid Ltd., Basingstoke, UK). Isolates showing inhibition zones ≤19 mm were confirmed by the penicillin Etest (AB Biodisk, Dalvagen, Solna, Sweden). Breakpoints used for interpretation of minimum inhibitory concentrations (MICs) were ≤0.06 mg/L (susceptible), 0.1–1.0 mg/L (intermediate) and ≥2 mg/L (resistant). *Streptococcus pneumoniae* ATCC 49619 was used as a reference strain for quality control.

### Serogrouping

Of the 162 *S. pneumoniae* strains recovered, 151 were available for serogrouping; the remaining 11 strains could not be recovered from stock culture at −20 °C. Strains were serotyped by Quelling reaction using 12 pooled Pneumotest antisera covering vaccine serotypes (Staten Serum Institute, Copenhagen, Denmark). For better visualisation, 1% methylene blue was used.

### Statistics

Chi-square, Yates-corrected and Fishers’ exact tests were used for assessment of carriage, clustering rates and serogroup distribution between the two study groups. A *P*-value of <0.05 was considered to be statistically significant.

## RESULTS

A single swab was obtained over the nasopharyngeal walls of the 500 apparently healthy children. Twenty five (5%) were found to be carriers (Table 1). The carrier rate was significantly higher in the seven-nine years-old age group. Initially, a questionnaire was given to the parents or guardians about some potential risk factors that may affect *S. pneumoniae* carriage. Upper respiratory tract infection within the last month (OR=1.1, p>0.001), day-care attendance (OR=3.1, p: 0.02), and the presence of more than five people living in the house of the child (OR=0.2, p: 0.003) have been determined to be risk factors for *S. pneumoniae* carriage. The most prevalent serogroups in descending order were 9, 19, 23, 6,10 and 18 and these are in accordance with conjugated pneumococcal vaccine (PCV7) (Table 2). No penicillin-resistant *S. pneumonia* were obtained.

**Table 1:** Total case number and distribution of carriage rate

	0–3 years old	4–6 years old	7–9 years old	10–12 years old	Total

Case number	125	125	125	125	500
Pnomococ carriage	0	9	16	0	25
Carriage rate %	% 0	% 7.2	% 10.8	% 0	% 5

**Table 2:** Distribution of serotypes

	Groups				

	0–3 years old	4–6 years old	7–9 years old	10–12 years old	Total number

Serotype 6	-	1 (% 4)	2 (% 8)	-	3 (% 12)
Serotype 9	-	3 (% 12)	7 (% 28)	-	10 (% 40)
Serotype 10	-	1 (% 4)	-	-	1 (% 4)
Serotype 18	-	1 (% 4)	-	-	1 (% 4)
Serotype 19	-	3 (% 12)	4 (% 16)	-	7 (% 28)
Serotype 23	-	-	3 (% 12)	-	3 (% 12)
Total	-	9 (% 36)	16 (% 64)	-	25 (% 100)

## DISCUSSION

Various factors may affect the nasopharyngeal colonization with *S. pneumoniae* including living conditions, season, respiratory illness and genetic traits (5, 17, 19). In our study, among a dozen possible risk factors, upper respiratory tract infection within the last month, day-care attendance, and the presence of more than five people living in the house of the child were confirmed as risk factors. It is known that *S. pneumoniae* carriage is more frequent in institutional environments such as day-care centers and crowded environment (11, 13, 17). Diseases due to *S. pneumoniae* frequently occur following mucosal damage, epithelial ciliary activity diminution and viral respiratory tract infections inhibiting function of alveolar macrophages (20). In this respect, a previous URTI may be a predisposing factor for increased nasopharyngeal colonization in our study population. In our study, breast-feeding and exposure to passive smoking were not associated with the *S. pneumoniae* carriage. The data regarding breast-feeding are still controversial regarding protection against respiratory pathogens and further studies are necessary (1, 6). Although smoking has been demonstrated as a risk factor for respiratory infections or colonization with nasopharyngeal pathogens in elderly, the role of passive smoking remains unexplored (9, 10). The overall rate of nasopharyngeal *S. pneumoniae* carriers varies among different populations. In a study from Rome, the carriage rate was found to be 14.9%, and living with more than three persons in the same household was reported to be the only risk factor statistically associated with carriage (14). Higher rates (19.4% and more than 75%) were also found in daycare centers in which many children stay together for a long periods (4) and (18). In this study, carriage rate was found as 5% in 500 healthy children and carriage rate was highest in 7-9 years old age group. In group 0-3 and 10-12 years-old, no isolates were found and totally no penicillin resistant isolates were found in this study, this is not in accordance with most studies about this subject.

In this study, the most prevalent serogroups in descending order were 9, 19, 23, 6, 10 and 18 and these are in accordance with conjugated pneumococcal vaccine (PCV7). Serogrouping is of paramount importance in planning new strategies for vaccine development, which can decrease and prevent the spread of pneumococcal infections and reduce mortality (12). In many countries, heptavalent pneumococcal vaccines containing serotypes 4, 6B, 9V, 14, 18C, 19F and 23F have been used and are effective in preventing invasive and non-invasive pneumococcal infections by reducing the carriage rate of antibiotic-resistant strains (7). Serotypes found in this study may help to prepare an appropriate pneumococcal vaccine in Turkey. After informing parents intensively and using intensive conjugated vaccine by campaigns, we think that vaccine has decreased carriage rates, particularly in children less than 5 years old. At the end, in Turkey, the health ministry decided to add this vaccine into routine vaccine programme. As a result, it is appears that carriage rate, penicillin resistant isolates, and infections will be significantly decreased by this vaccine in the near future.
